# Effect of TACI Signaling on Humoral Immunity and Autoimmune Diseases

**DOI:** 10.1155/2015/247426

**Published:** 2015-03-17

**Authors:** Yi Zhang, Jun Li, Ya-Min Zhang, Xiao-Ming Zhang, Juan Tao

**Affiliations:** Department of Dermatology, Affiliated Union Hospital, Tongji Medical College, Huazhong University of Science and Technology, Wuhan 430022, China

## Abstract

Transmembrane activator and calcium-modulating cyclophilin ligand interactor (TACI) is one of the receptors of B cell activating factor of the tumor necrosis factor family (BAFF) and a proliferation-inducing ligand (APRIL). TACI is a regulator in the immune responses. TACI inhibits B cell expansion and promotes the differentiation and survival of plasma cells. The mechanisms underlying these effects probably involve changed expressions of some crucial molecules, such as B lymphocyte induced maturation protein-1 (Blimp-1) and inducible T-cell costimulator ligand (ICOSL) in B cells and/or plasma cells. However, abnormal TACI signaling may relate to autoimmune disorders. Common variable immune deficiency (CVID) patients with heterozygous mutations in *TACI* alleles increase susceptibility to autoimmune diseases. *Taci* ^−/−^ mice and BAFF transgenic mice both develop signs of human SLE. These findings that indicate inappropriate levels of TACI signaling may disrupt immune system balance, thereby promoting the development of autoimmune diseases. In this review, we summarize the basic characteristics of the TACI ligands BAFF and APRIL, and detail the research findings on the role of TACI in humoral immunity. We also discuss the possible mechanisms underlying the susceptibility of CVID patients with *TACI* mutations to autoimmune diseases and the role of TACI in the pathogenesis of SLE.

## 1. Introduction

Belimumab, a specific inhibitor of B cell activating factor (BAFF), was approved in 2011 by the US Food and Drug Administration (FDA) for the treatment of systemic lupus erythematosus (SLE). The FDA approval of belimumab not only represents the significant progress in the field of SLE therapeutics but also marks the success of BAFF research. BAFF and its homologue, a proliferation inducing ligand (APRIL), are recently discovered members of the tumor necrosis factor (TNF) superfamily [1]. BAFF and APRIL interact with three specific receptors, calcium modulator and cyclophilin ligand interactor (TACI), B cell maturation antigen (BCMA), and BAFF receptor (BAFF-R or BR3), thereby constituting a complex system. The system plays a variety of roles in immunomodulation, mainly by affecting B cell activation, proliferation, and survival. BR3 only binds to BAFF, and the primary role of BR3 is to mediate the survival and maturation of peripheral B cells. Both BCMA and TACI are capable of binding to BAFF and APRIL. BCMA is primarily expressed in plasma cells, and its primary role is to mediate the survival of long-lived bone marrow plasma cells [1].

TACI is a regulator that affects multiple events in the immune responses. Firstly, TACI inhibits B cell expansion [2, 3]. Secondly, TACI induces IgG and IgA class switch recombination in B cells. Finally, TACI promotes the differentiation and survival of plasma cells [4–6]. How TACI exerts its effects remains unclear; however, several recent studies provide relatively reasonable explanations [4–6]. Additionally, abnormal TACI signaling may relate to autoimmune disorders. For example,* Taci* ^−/−^ mice and BAFF transgenic mice both develop symptoms of SLE-like autoimmune diseases [7–10], which indicates that although a loss of TACI signaling contributes to the incidence of autoimmune diseases, an increase in TACI signaling due to elevated BAFF level fails to prevent the occurrence of autoimmune diseases. Furthermore, although* TACI* mutations are associated with common variable immunodeficiency (CVID) patients, heterozygous mutations and homozygous mutations in* TACI* alleles have entirely different effects on incidence of autoimmune diseases [11–13]. Therefore, whether TACI plays an autoimmune disease-promoting or an autoimmune disease-inhibiting role remains to be elucidated.

In the present review, we summarize the basic characteristics of the TACI ligands BAFF and APRIL and detail the research findings on the role of TACI in B cells and humoral immunity. We also discuss the possible mechanisms underlying the susceptibility of CVID patients with* TACI* mutations to autoimmune diseases and the role of TACI in the pathogenesis of SLE.

## 2. The Basic Characteristics of the TACI Ligands BAFF and APRIL

### 2.1. BAFF

BAFF is a type II transmembrane protein that belongs to the TNF ligand superfamily. BAFF is mainly produced by myeloid cells, such as monocytes, macrophages, neutrophils, and dendritic cells (DCs) [1]. Radioresistant stromal cells, activated T cells, B cells, and certain nonhematopoietic cells in bone marrow are also capable of producing BAFF and APRIL [14, 15]. Goenka et al. [16] reported that BAFF is mainly produced by follicular helper T cells (T_FH_) in the germinal center (GC). T_FH_-derived BAFF plays an important role in the survival of high-affinity B cell clones.

A variety of cytokines, including interferon gamma (IFN-*γ*), transforming growth factor beta (TGF-*β*), and interferon alpha (IFN-*α*), are capable of upregulating the expression of BAFF on myeloid cells [17]. Additionally, estrogen also upregulates the expression of BAFF [18], which is consistent with the phenomenon that a variety of autoimmune diseases occur more frequently in females. However, increased BAFF expression does not always correlate with increased BAFF secretion. It has been shown that activation of toll-like receptor 9 (TLR9) upregulates the expression of membrane-bound BAFF in human B cells but fails to alter the peripheral BAFF concentration [19], indicating that the BAFF expressed on the B cell surface is not secreted in large amounts. Although various factors are capable of upregulating BAFF expression, distinct intracellular signaling pathways are involved [20, 21]. Therefore, it is extremely difficult to suppress BAFF at the cellular level because the simultaneous blockage of multiple, distinct signaling pathways is required.

BAFF exists in two forms, a membrane-bound form and a soluble form. Membrane-bound BAFF is cleaved at a furin protease site, releasing soluble BAFF [19]. Soluble BAFF mainly exists in the form of homotrimers. An* in vitro* study has shown that 20 BAFF trimers may associate to form a BAFF 60-mer, which exhibits a virus-like structure, at a neutral or alkaline pH. At an acidic pH, the BAFF 60-mer dissociates into BAFF trimers [22]. However, whether soluble BAFF does or does not form BAFF 60-mer* in vivo* is controversial [17].

The B cell numbers and immune responses in mice expressing BAFF with a mutated furin protease cleavage site are similar to those in BAFF-deficient mice, indicating that BAFF primarily exerts its effects in the form of soluble BAFF (including the trimer and 60-mer forms) [23]. Membrane-bound BAFF and soluble BAFF work together to regulate the expression of cluster of differentiation 23 (CD23) in B cells [23]. Additionally, membrane-bound BAFF exerts a relatively weak effect on the production and survival of B2 B cells in the peritoneal cavity, the differentiation of marginal zone (MZ) B cells, and the production of basal levels of immunoglobulin A (IgA) [23]. However, in mice expressing BAFF with a mutated furin protease cleavage site, expression of membrane-bound BAFF was much lower than that in wild type (WT) mice. Therefore, the observed phenomena in the engineered mice may be due to insufficient expression of membrane-bound BAFF rather than biological effects of membrane-bound BAFF itself [17]. Recently, an* in vitro* study demonstrated that both membrane-bound BAFF and soluble BAFF promote the proliferation of human B cells in the presence of anti-immunoglobulin M (IgM) antibodies [19].

Delta-BAFF, an isoform of BAFF, may play a role in regulating BAFF activity [24]. Compared with BAFF, delta-BAFF lacks a short peptide segment in its structure. As a result, delta-BAFF is unable to bind to cell-surface BAFF receptors. Instead, delta-BAFF interacts with full-length BAFF to form inactive trimers, decreasing the level of bioactive full-length BAFF [24].

### 2.2. APRIL

APRIL is also a type II transmembrane protein that belongs to the TNF ligand superfamily. APRIL is mainly produced by myeloid cells. Certain nonhematopoietic cells and tumor cells also produce APRIL [1]. APRIL differs from BAFF in that APRIL is not present on the cell surface. APRIL is processed by the Golgi apparatus, which involves cleavage at the Furin protease site, and the resulting soluble APRIL is released from the cell [25]. There are, however, exceptions. For example, Maia et al. [26] reported that an APRIL isoform lacking the furin protease cleavage site, APRIL-*δ*, is expressed on the surface of leukemia cells. Another exception is the TWE-PRIL protein, which is expressed on the surface of T cells and monocytes and is also known as the tumor necrosis factor (ligand) superfamily member 12-member 13 (TNFSF12-TNFSF13). TWE-PRIL is a hybrid protein between APRIL and TNF-related weak inducer of apoptosis (TWEAK, also known as TNFSF12) and is derived from the trans-splicing of the adjacent APRIL and TWEAK genes [27].

Similar to soluble BAFF, soluble APRIL mainly exists in the form of homotrimers. APRIL trimers are unable to form a 60-mer. However, APRIL trimers bind to heparan sulfate proteoglycans (HSPGs), and the binding of multiple APRIL to HSPGs enhances local APRIL signaling [28]. Moreover, HSPGs provide a platform for APRIL multimerization, which promotes the occurrence of APRIL multimerization [29]. Additionally, a study has shown that APRIL plays an important role in maintaining the number of B1 B cells in the peritoneal cavity by binding to HSPGs rather than TACI [30]. However, the exact mechanism underlying this effect remains unclear.

Soluble APRIL forms a BAFF/APRIL heterotrimer with BAFF [31]. The concentration of the BAFF/APRIL heterotrimer is increased in rheumatoid arthritis (RA) and SLE [32]. An* in vitro* study has shown that the BAFF/APRIL heterotrimer has biological activities, but the BAFF/APRIL heterotrimer activity is weaker than the activity of the BAFF homotrimer [32]. It is possible that the BAFF/APRIL heterotrimer activity* in vivo* is also weaker compared with the BAFF homotrimer activity. BAFF/APRIL heterotrimers may compete with BAFF homotrimers for BAFF receptors* in vivo*, which most likely reduces the overall activity of BAFF [33]. Therefore, the increased BAFF/APRIL heterotrimer concentrations in SLE and RA may represent a regulatory feedback mechanism used by the organism in response to the elevated levels of BAFF.

### 2.3. The Forms of Ligands to Activate TACI

**Figure 1:**
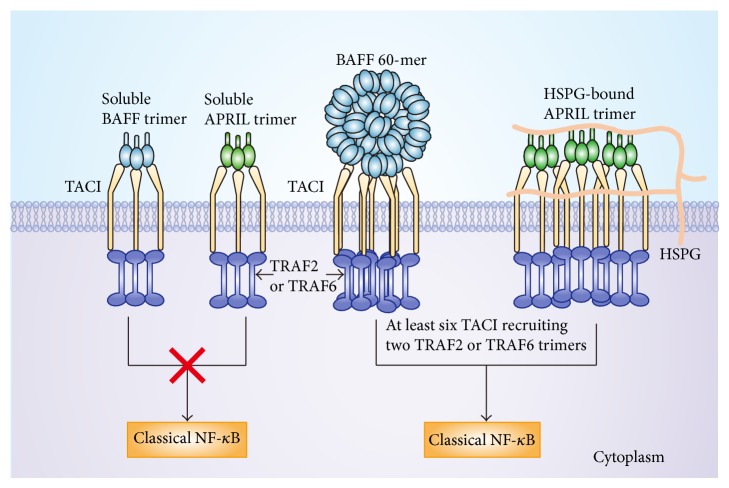
The forms of ligands to activate TACI. Both the BAFF 60-mers and multimerized APRIL are able to activate TACI; however, BAFF trimers and APRIL trimers cannot activate TACI. Because at least two TRAF trimers need to associate six TACIs to activate the classical NF-*κ*B pathway.

Although the trimer forms of the ligands (BAFF trimer or APRIL trimer) are capable of binding to TACI, the trimers lack the ability to activate TACI [34]. The inability of the trimers to activate TACI may be because signal transduction from TACI first requires binding to tumor necrosis factor receptor-associated factor (TRAF) 2 or TRAF6 in the cell. TRAFs exist in the form of trimmers, and a single TRAF trimer has low affinity for TACI. To activate the nuclear factor-kappa B (NF-*κ*B) pathway, two TRAF trimers need to associate with at least six TACIs, which produces avidity effects and significantly strengthens the binding force between TACI and TRAF (Figure 1) [35, 36]. Consistent with the above-mentioned results, both the BAFF 60-mer and multimerized APRIL are able to activate TACI [28, 29].

HSPGs bind to APRIL and TACI and play an important role in activating the APRIL-TACI pathway [28]. For example, syndecan-1 forms complexes with APRIL and TACI, which promote activation of the APRIL-TACI pathway and thereby induce the survival and proliferation of multiple myeloma cells. Furthermore, heparanase (which is capable of degrading HSPGs) efficiently suppresses these effects [37]. The mechanism of action of HSPGs may involve providing a platform for APRIL multimerization and enhancing the binding of APRIL to TACI. As a result, HSPGs promote activation of the APRIL-TACI pathway.

## 3. The Biological Activities of TACI

### 3.1. TACI Expression

In mice, TACI is mainly expressed by mature B cells. High TACI expression has been detected in innate immune B cells, such as MZ B cells and B1 B cells [38]. In humans, TACI is mainly expressed by CD27^+^ memory B cells, tonsillar and bone marrow plasma cells, a portion of activated CD27^−^ non-GC cells, monocytes, DCs, and a portion of naive B cells in the blood plasma and tonsils [38]. Additionally, TACI has also been reported to be expressed in macrophages and mediate macrophage survival [39].

**Figure 2:**
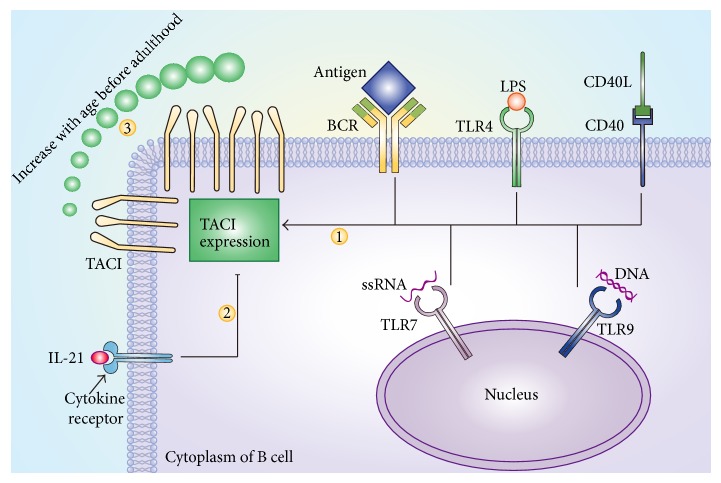
Regulation of TACI expression. Activation TLR4, TLR7, or TLR9 increases TACI expression in B cells. Ligation of CD40 or BCR activation also results in elevated TACI expression in B cells (1). However, IL-21 downregulates TACI expression in GC B cells (2). TACI expression in B cells increases with age before adulthood (3).

The TACI expression level is regulated by a variety of factors and constantly exhibits dynamic changes. For example, activation of TLR9, TLR7, or TLR4 results in elevated TACI expression in B cells [21, 40–42]. A study conducted by Treml et al. [42] showed that the TLR4 activation-induced increase in TACI expression is dependent on myeloid differentiation primary response protein 88 (MyD88) and c-Rel. In contrast, the TLR9 activation-induced increase in TACI expression depends on MyD88 but not c-Rel. CD40 ligand (CD40L) is also capable of upregulating TACI expression in B cells [43], and interleukin-21 (IL-21) downregulates TACI expression in GC B cells [16]. Furthermore, TACI expression is reduced in mice with X-linked immunodeficiency (XID), which is likely due to the impaired B cell receptor (BCR) signaling caused by defects in Bruton's tyrosine kinase (Btk) [21]. These results indicate that BCR activation is involved in regulating TACI expression (Figure 2).

TACI expression level is related to age. Schatorjé et al. [44] found that the percentage of TACI^+^ B cells is positively correlated with age in adolescents. TACI expression is reduced in the cord blood B cells of premature babies [45]. In newborns, TACI, BCMA, and APRIL are expressed at rather low levels, resulting in a lack of IgA-secreting plasma cells. Therefore, newborns are susceptible to certain intestinal bacteria [46]. Newborn mice express a considerably low level of TACI, and plasma cell production and antibody secretion are significantly reduced in newborn mice compared with adult mice. Stimulation of newborn mouse-derived B cells with deoxycytidyl-deoxyguanosine oligodeoxynucleotides (CpG ODN) results in increased TACI expression, and the plasma cell production and antibody secretion levels are similar to the levels in adult mice [47]. However, since TACI is highly expressed by MZ B cells and B1 B cells in mice and newborn mice have no MZ B cells [1], TACI expression increases with age before adulthood may simply reflect the establishment of MZ B cells.

Although TACI is mainly present on the cell surface, a small amount of TACI is shed from the cell surface by a disintegrin and metalloproteinase (ADAM) 10 and converted into soluble TACI (sTACI) [38, 48]. The sTACI plasma levels are elevated in SLE, chronic lymphocytic leukemia (CLL), and CVID patients [49, 50]. Additionally, in SLE and CLL patients, the sTACI levels are related to disease activities. The sTACI functions remain unclear, but it is likely that sTACI antagonizes the binding of BAFF or APRIL to their cell surface receptors. Considering that the plasma level of BAFF is elevated in patients with SLE, CVID, and CLL, the increased sTACI level may be related to feedback mechanisms by which the organism regulates the degree of BAFF activity.

### 3.2. TACI Regulates the Number of Peripheral B Cells

*In vitro* studies have shown that activation of TACI decreases the numbers of human and mouse B cells [2, 3]. Furthermore,* Taci* ^−/−^ mice develop hyperplasia in lymphoid organs, and the number of B cells increases drastically, indicating that TACI also exerts an inhibitory effect on B cells* in vivo* [3–5].

TACI partially inhibits B cell number by promoting the sustained expression of B lymphocyte induced maturation protein-1 (Blimp-1) in GC B cells [6]. Blimp-1 is a transcriptional repressor that is capable of inducing cell cycle arrest in B cells and promoting the differentiation of B cells into plasma cells. Blimp-1 partially exerts its effect by inhibiting B cell lymphoma 6 (BCL-6) [6].

Additionally, the expression of inducible T-cell costimulator ligand (ICOSL) is enhanced in the GC B cells of* Taci* ^−/−^ mouse, which strengthens the costimulatory signals received by inducible T-cell costimulator (ICOS) on T_FH_ cells and promotes the activation and proliferation of T_FH_ cells. The increase in the T_FH_ number enhances the auxiliary effect of T_FH_ cells on GC B cells and promotes B cell activation and proliferation, thereby increasing the number of B cells [5]. These findings indicate that TACI may inhibit GC reactions and B cell proliferation by suppressing the expression of ICOSL on GC B cells.

The inhibitory effect of TACI on the B cell number is partially achieved through promoting B cell apoptosis. Tsuji et al. [4] reported that TACI promotes the apoptosis of GC B cells and upregulates the expression of the cellular inhibitor of apoptosis protein (cIAP) in GC B cells. cIAP targets NF-*κ*B-inducing kinase (NIK) for degradation by ubiquitin-proteasome system, thereby inhibiting the BR3-mediated noncanonical NF-*κ*B signaling pathway [51]. The promotion of B cell apoptosis by TACI may be related to activation induced cell death (AICD). In the humoral immune responses to TD antigens, AICD exerts its effect mainly through the interaction between the Fas ligand (FasL) on the T cell surface and Fas on the B cell surface. In the humoral immune responses against TI antigens, the synergistic effect of TACI and TLR4 leads to the simultaneous increase in the expression of FasL and Fas on the surface of MZ B cells, thereby promoting AICD [52].

It is noteworthy that TACI may exert its inhibitory effect indirectly by regulating the BAFF concentration. The plasma BAFF level is significantly higher in* Taci* ^−/−^ mice compared with WT mice, which may be because the B cells in* Taci* ^−/−^ mice are unable to bind to BAFF through TACI and reduce the concentration of BAFF [16, 34, 53].

### 3.3. The Role of TACI in Somatic Hypermutation and Antibody Class Switching

Somatic hypermutation of immunoglobulin (Ig) genes is not only one of the mechanisms leading to antibody diversity, but it is also the main mechanism underlying Ig affinity maturation. A study conducted by Tsuji et al. [4] showed that the GC B cells in* Taci* ^−/−^ mice undergo an increased number of cell cycles resulting in an increased level of somatic hypermutation. A high degree of somatic hypermutation causes an increase in the number of B cell clones that express high-affinity BCR, which eventually results in the production of antigen-specific antibodies with increased affinity in* Taci* ^−/−^ mice compared with WT mice. These results indicate that TACI exerts a regulatory effect on somatic hypermutation and Ig affinity maturation.

Antibody class switch recombination (CSR) is another mechanism that leads to antibody diversity. Mouse studies have demonstrated that TACI mediates T cell-independent CSR [9, 47, 54]. TACI mediates CSR by binding to MyD88 through the TACI highly conserved (THC) region, thereby activating the classical NF-*κ*B pathway, prompting the transcription of germline *C*_*H*_ genes, and inducing the expression of activation induced cytidine deaminase (AID) [55].

TACI alone or TLRs alone are able to mediate T cell-independent CSR by binding to MyD88. TACI-mediated CSR does not require TLRs, but both TLR7 and TLR9 together with TACI have a synergistic effect in promoting CSR [55]. Similarly, CD40, a protein that mediates T cell-dependent CSR, also exhibits a synergistic effect with TACI in promoting CSR [56]. Furthermore, BCR activation alone is unable to mediate CSR, but BCR activation promotes TLR- or TACI-mediated CSR [57]. The mechanisms underlying the synergistic or promoting effects may involve the simultaneous activation of the classical and noncanonical NF-*κ*B pathways and/or the upregulation in TACI expression [43, 47, 57, 58].

In summary, it has been shown that TACI plays a role in both T cell-dependent and T cell-independent CSR by binding to MyD88. Consistent with these findings, mutations in the gene encoding THC obliterate the synergistic effect of TACI and CD40 and the synergistic effect of TACI and TLRs on inducing CSR [59].

### 3.4. The Role of TACI in Plasma Cell Differentiation and Antibody Production

TACI plays an important role in the antibody response to TI-2 antigens. The antibody response to TI-2 antigens is mainly mediated by MZ B cells and B1 B cells, and TACI is expressed at the highest levels in these two B cell subsets [41, 52]. Additionally,* Taci* ^−/−^ mice fail to produce an effective antibody response against TI-2 antigens [9, 10]. The antibody response to TI-1 antigens also requires TACI. Upon stimulation with the TI-1 antigen 2, 4, 6-trinitrophenol- (TNP-) lipopolysaccharide (LPS), Taci^−/−^ mice produce the IgM and immunoglobulin G1 (IgG1) antibodies at significantly lower levels compared with WT mice [60].

Additionally, TACI also plays a role in the immune response to TD antigens. Castigli et al. [43] reported that TACI promotes the cluster of differentiation 40- (CD40-) mediated plasma cell differentiation and antibody production, indicating that TACI promotes TD antigen-induced immune responses. However, a study conducted by Sakurai et al. [2] showed that TACI inhibits CD40- and BR3-mediated antibody production, indicating that TACI has an inhibitory effect on TD antigen-induced immune responses.

It should be noted that these two* in vitro* studies described above were conducted under very different conditions. In the former study, mouse naive B cells were used and CD40 was stimulated with less than the optimal concentration of anti-CD40 antibody. In the latter study, human peripheral blood B cells were used and CD40 was allowed to interact with a relatively high concentration of soluble CD40 ligand (CD40L). The discrepancy between these two studies may be due to the difference in the CD40 signal strength and/or differences in the effect of TACI in the mouse and human.

Several recent studies have demonstrated that antibody concentrations are significantly lower following TD antigen stimulation in* Taci* ^−/−^ mice compared with WT mice [4–6]. These results indicate that TACI promotes the immune responses against TD antigens. Tsuji et al. [6] reported that the titers of antigen-reactive antibodies are reduced in* Taci* ^−/−^ mice. These decreased antibody titers are caused by defective Blimp-1 expression in the GC B cells, which inhibits differentiation toward plasma cells and results in a decreased number of plasma cells.

In the early stages of the immune responses against influenza virus, the humoral immune responses generated in the* Taci* ^−/−^ mice are similar to those generated in the WT mice. However, in the late stages of the immune responses, number of antibody-producing cells (ASCs) and the plasma antibody titers are significantly lower in the* Taci* ^−/−^ mice compared with the WT mice [61]. It is possible that the GC reaction is enhanced in* Taci* ^−/−^ mice during the early stages of the immune response. The number of DNA double-strand breaks increases accordingly, leading to a transient expression of Blimp-1 [6]. Therefore, the humoral immune responses in the Taci^−/−^ mice appear normal. However, sustained Blimp-1 expression requires TACI activity. Therefore, the levels of ASCs and antibodies cannot be maintained in* Taci* ^−/−^ mice during the late stages of the immune response.

Ou et al. [5] also demonstrated that the titers of antigen-reactive antibodies are reduced in* Taci* ^−/−^ mice. However, the reduction in antibody titers is due to increased plasma cell apoptosis. The proapoptotic protein B cell lymphoma 2 interacting mediator of cell death (BIM) promotes apoptosis in plasma cells. APRIL and BAFF downregulate BIM expression by interacting with TACI, thereby promoting the survival of plasma cells.* Taci* ^−/−^ mice are unable to downregulate BIM expression through APRIL and BAFF, resulting in increased plasma cell apoptosis.

**Figure 3:**
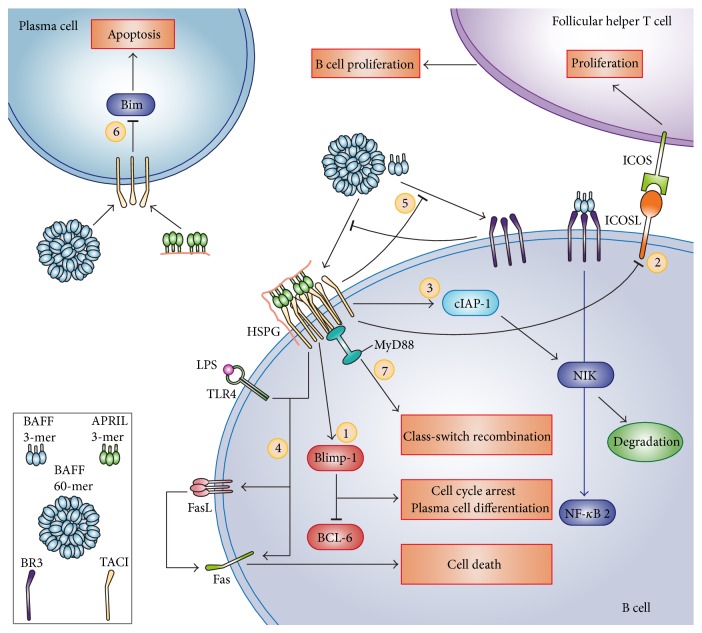
The biological activities of TACI. TACI inhibits B cell proliferation by promoting the sustained expression of Blimp-1. Blimp-1 induces cell cycle arrest in B cells, promoting the differentiation of B cells into plasma cells (1). TACI inhibits GC reactions and B cell proliferation by suppressing the expression of ICOSL on GC B cells (2). TACI upregulates the expression of cIAP in GC B cells. cIAP targets NIK for degradation by ubiquitylation, thereby inhibiting the BR3-mediated noncanonical NF-*κ*B pathway (3). TACI and TLR4 signalling cooperate to trigger MZ B cells to apoptosis by induction of Fas and FasL (4). TACI competes with BR3 for BAFF, which also downregulates the BAFF concentration (5). TACI inhibits plasma cell apoptosis by downregulating BIM expression in plasma cells (6). TACI mediates CSR by binding to MyD88 (7).

The two studies described above indicate that TACI regulates the number of plasma cells and the production of antibodies through at least two mechanisms. First, TACI promotes the differentiation of B cells into plasma cells by maintaining a continuous expression of Blimp-1 in B cells. Second, TACI inhibits plasma cell apoptosis by downregulating BIM expression in plasma cells (Figure 3). Higher plasma cell numbers are correlated with higher antibody titers, while lower plasma cell numbers are correlated with lower antibody titers.

### 3.5. Reverse Signaling of TACI

Studies have shown that TACI binds to membrane-bound ligands and activates macrophages or DCs through reverse signaling [62–64]. For example, a study conducted by Diaz-de-Durana et al. [65] demonstrated that TACI-expressing B cells interact with BAFF-expressing DCs through TACI-BAFF, which promotes the development of DCs into antigen-presenting cells capable of efficiently activating T cells. The role of B cells in DC development may be replaced by TACI-Fc fusion protein. An alternative explanation has been proposed. Because TACI is capable of binding to HSPGs and HSPGs can activate DCs through TLR4, it is possible that TACI indirectly activates DCs by promoting the effect of HSPGs on TLR4 [66–68].

A recent study has shown that there is no difference in the cellular responses between BAFF and APRIL knockout cells and WT cells under the activity of soluble TACI-Fc or soluble BR3-Fc [69]. Therefore, this study fails to confirm the existence of TACI-BAFF/APRIL reverse signaling. Additionally, the study also demonstrated that only multimeric TACI-Fc is capable of activating macrophages and that the TACI-Fc multimer is unable to activate macrophages that lack the Fc receptor *γ*-chain (FcR*γ*) on their surface. Therefore, the activating effect of TACI-Fc previously observed in DCs and macrophages is likely induced by the activating signals that are generated via the high-molecular-weight TACI-Fc multimer-mediated cross-linking of Fc receptors [69]. Because only macrophages were examined in the study, it remains unclear whether TACI-BAFF/APRIL reverse signaling occurs in DCs and other types of cells.

## 4. Abnormal TACI Signaling and Autoimmune Diseases

### 4.1. CVID-Associated TACI Mutations Affect Susceptibility to Autoimmune Diseases

Approximately 7% to 10% of CVID patients carry allelic mutations in the* TACI* gene, primarily the heterozygous C104R and A181E mutations [40, 70, 71]. The heterozygous* TACI* mutations C104R and A181E are also found in approximately 2% of healthy individuals [12]. Although the individuals show no clinical manifestations of CVID, defects in B cell functions have been detected using* in vitro* assays. For example, compared with healthy individuals who do not carry* TACI* gene mutations, individuals with heterozygous* TACI* mutations demonstrate decreased TACI expression on their B cells. Additionally, stimulation of B cells with TLR9 fails to upregulate the expression of TACI, and stimulation of TACI with activating antibodies is unable to effectively induce the expression of AID mRNA in B cells [12].

However, the decrease in TACI expression and defects in TACI functions do not always have a negative impact on the immune system. Tsuji et al. [4] demonstrated that although the baseline Ig levels are lower in* Taci* ^−/−^ mice compared with WT mice, the* Taci* ^−/−^ mice are able to produce antigen-specific antibodies with a higher affinity for the antigens and are able to clear* Citrobacter rodentium* at a faster rate. The results indicate that the high frequencies of monoallelic or biallelic* TACI* mutations in human populations may be an adaptation to the environment. Defects in TACI expression and function may allow part of the human population to rapidly produce high-affinity IgG antibodies to clear intestinal pathogens. However,* TACI* mutations increase the risk of developing CVID [12].

Additionally,* TACI* mutations in CVID correlate with susceptibility to autoimmune disorders. TACI regulates the function of BCR, TLR7, and TLR9. BCR, TLR7, and TLR9 may be involved in self-antigen recognition and central B cell tolerance [72]. Therefore,* TACI* gene mutations impair central B cell tolerance, resulting in an inability to remove autoreactive B cells through central tolerance mechanisms [11]. In healthy individuals that carry the mutated* TACI* alleles, normal peripheral tolerance may compensate for defects in central tolerance. Therefore, these individuals are not prone to autoimmune diseases. CVID patients, however, exhibit abnormalities in peripheral tolerance mechanisms. The level of BAFF is elevated in CVID patients, and the level of regulatory T cells (Treg) is reduced [49, 73, 74]. In CVID patients that carry allelic mutations in the* TACI* gene, peripheral tolerance is unable to compensate for defects in central tolerance. Therefore, CVID patients are susceptible to autoimmune diseases [11].

Although heterozygous mutations in* TACI* alleles are related to a high level of T_FH_ and high titers of IgG antinuclear antibodies, the level of T_FH_ is extremely low in CVID patients that are homozygous for mutant* TACI* alleles. Moreover, IgG antinuclear antibodies are undetectable in these patients [11]. These phenomena may be due to impaired ICOSL expression in B cells with homozygous mutations in* TACI* alleles. Impaired ICOSL expression leads to a loss of interactions between the ICOSL on B cells and the ICOS on T_FH_ cells. As a result, both the B cells and T_FH_ cells are unable to undergo efficient activation and proliferation, and GCs fail to form [75–77]. In* Taci* ^−/−^ mice, however, the expression of ICOSL on B cells is not reduced and the number of T_FH_ cells is increased [4], indicating that further studies are required to clarify the differences in TACI function between species.

Additionally, TACI promotes the differentiation and survival of plasma cells [4–6]. Therefore, homozygous mutations in* TACI* alleles result in a complete loss of TACI function and an inability to maintain continuous production of autoreactive antibodies. In contrast, heterozygous mutations in* TACI* alleles only impair TACI function. TACI function is not completely lost and is sufficient to maintain the continuous production of autoreactive antibodies [4]. Therefore, CVID patients that are heterozygous for mutant* TACI* alleles are prone to autoimmune diseases, whereas CVID patients homozygous for mutant* TACI* alleles appear to experience an autoimmune disease-preventive effect [11–13].

As* Taci* ^−/−^ mice age, they display SLE-like symptoms [3]. Additionally, loss of the* TACI* gene is not sufficient to prevent the incidence of SLE in NZM2328 mice [78]. Therefore, the proposal that a complete loss of TACI function may prevent autoimmune diseases appears to only apply to patients with CVID. Further studies need to be conducted to solve the mystery surrounding TACI.

### 4.2. TACI and SLE: An Overly Strong or Very Weak TACI Signal Is Not Beneficial

BAFF transgenic mice exhibit SLE-like symptoms [7]. Blockage of BAFF attenuates the clinical symptoms in lupus mice [8]. In human SLE patients, plasma BAFF concentrations may increase by 50% [79–81]. A number of studies have shown that the plasma concentration of BAFF is related to SLE disease activity [82, 83]. Belimumab, a specific BAFF antagonist, has successfully passed clinical trials and has been approved by the US FDA for treating SLE [84, 85]. The role of BAFF in the pathogenesis of SLE has been defined. However, whether TACI exerts a promoting or inhibitory effect on SLE pathogenesis remains unclear.

The number of B cells is increased in* Taci* ^−/−^ mice. As* Taci* ^−/−^ mice age, the mice develop SLE-like symptoms [3, 9, 10]. These findings not only demonstrate that TACI is not necessary for SLE pathogenesis but also indicate that TACI may inhibit SLE pathogenesis. No significant difference has been detected in the incidence of SLE between* Taci* ^−/−^ NZM2328 and WT NZM2328 mice. However, the renal histology scores are significantly higher in 5-month-old and 8-month-old* Taci* ^−/−^ NZM2328 mice compared with the corresponding WT NZM2328 mice [78], which supports an inhibitory effect of TACI on SLE.

Additionally, the increased number of GC B cells in* Taci* ^−/−^ mice is mainly due to increased B cell proliferation [5, 6]. When the mutation rate remains unchanged, the number of mutations increases exponentially as the cells proliferate [86]. Therefore, an increase in GC B cells proliferation results in an increased number of autoreactive B cells, which also supports an inhibitory effect of TACI on SLE.

Moreover, TACI promotes the expression of AID [55]. A study conducted by Chen et al. [87] showed that AID blocks disease aggravation in lupus mice. In humans, AID plays a vital role in the removal of autoimmune B cells [88]. These findings also demonstrate that TACI exerts an inhibitory effect on SLE.

However, other evidence suggests that the effect exerted by TACI on SLE is not simply inhibitory. There is no significant difference in the incidence of SLE between* Br3* ^−/−^ NZM2328 mice and WT NZM2328 mice. Although the numbers of follicular (Fo) B cells and MZ B cells are significantly reduced in* Br3* ^−/−^ NZM2328 mice, no difference has been found in the number of plasma cells between* Br3* ^−/−^ and WT NZM2328 mice [78]. The reason for these findings may be that an elevated level of BAFF enhances the TACI-mediated effects. TACI promotes plasma cell differentiation and survival [5, 6]. Therefore, even though the number of B cells is significantly decreased, the enhanced TACI signaling not only leads to an increase in the percentage of B cells that undergo differentiation into plasma cells but also may promote the survival of plasma cells. Thus, there is no difference in the number of autoantibody-secreting plasma cells between* Br3* ^−/−^ and WT NZM2328 mice [78]. These findings indicate that enhanced TACI signaling promotes SLE.

Additionally, distinct types of autoantibodies contribute differently to the pathogenesis of SLE [89]. Most of the pathological changes in SLE are related to IgG autoantibodies, whereas the concentration of the antidouble stranded DNA IgM is negatively correlated with the incidence of kidney damage in SLE patients [90]. In the female (NZB × NZW) F1 mouse model of lupus, the subcutaneous injection of monoclonal antidouble stranded DNA IgM attenuates renal damage and increases the survival time of the mice [91]. TACI mediates CSR by binding to MyD88 [55]. In SLE, TACI signal is too strong due to elevated BAFF level. Therefore, TACI will promote antibody class switching from autoreactive IgM to IgG, which may contribute to the aggravation of SLE.

**Figure 4:**
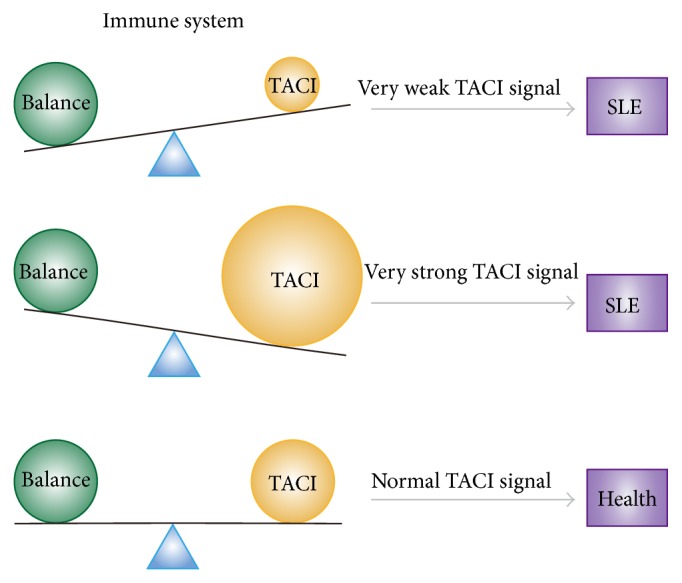
Conjectured relations between TACI signal intensity and immune system balance. Overly strong or very weak TACI signal will disrupt the immune system balance and promote SLE.

As described above, TACI may inhibit SLE, but an overly strong TACI signal may promote SLE. Will TACI promote or inhibit SLE then? Indeed, the so-called normal immune system is in a state of dynamic equilibrium, and autoimmune diseases are caused by an imbalance in the system. Therefore, we conjecture that TACI signaling at a normal intensity is conducive to maintaining the body's immune balance and exerts an inhibitory effect on SLE. However, too strong of a TAC1 signal is just as detrimental as too weak of a signal. An overly strong or very weak TACI signal will disrupt the immune system balance and promote SLE (Figure 4).

The characteristics of TACI signaling may partially explain the paradox concerning the efficacy of the monoclonal antibodies belimumab and rituximab in treating SLE. Belimumab was successful in 2 independent phase III SLE trials [84, 85]. However, rituximab demonstrated a stronger ability to remove B cells compared with belimumab but failed to achieve its end points in 2 independent phase II/III SLE trials [92, 93]. The reason for these effects may partially be due to the elevated plasma BAFF levels in SLE patients after treatment with rituximab. Under the enhanced TACI signaling, a higher percentage of the remaining autoreactive B cells differentiate into autoantibody-secreting plasma cells, thus promoting the development of SLE.

## 5. Conclusions

TACI plays an important role in the antibody responses to not only TI-2 antigens but also TI-1 and TD antigens. The mechanisms by which TACI regulates the number of B cells and promotes plasma cell differentiation and survival provide a rather reasonable explanation for TACI activity. Certainly, these mechanisms are not necessarily comprehensive. Further studies need to be conducted to fully elucidate the mechanisms regulating TACI function.

Based on the large amount of contradictory findings, it is impossible to conclude that TACI exerts a simple promoting or inhibitory effect on autoimmune diseases such as SLE. TACI is a factor that affects multiple events in the humoral immune responses. Therefore, the intensity and/or the background of TACI activity may determine the outcome. TACI signaling at an appropriate level suppresses autoimmunity and maintains immune balance and tolerance. In contrast, overly strong or very weak TACI signaling does not appear to be conducive to immune balance and tolerance and thus promote SLE.

However, the so-called “appropriate TACI signal strength” is different under different conditions. For example, homozygous mutations in* TACI* alleles exert a preventive effect on autoimmune diseases. Conversely, heterozygous mutations in* TACI* alleles result in a decrease rather than complete loss of TACI function and promote autoimmune diseases [13, 40]. If a complete loss of TACI function results in a failure to maintain continuous production of autoreactive antibodies, then the finding that* Taci* ^−/−^ mice develop SLE appears to be contradictory [3].

Certainly, TACI is just one of the various factors that affects autoimmune diseases. The roles of the other factors, such as T cells, cannot be ignored. The pathogenesis of SLE in BAFF transgenic mice does not depend on T cells [41], but it remains unknown whether T cells play a key role in the pathogenesis of SLE in the case of impaired TACI function. Studies using a combination of T cell-deficient and TACI-deficient mice may be able to address this question.
